# Bio-efficacy and wash-fastness of a lambda-cyhalothrin long-lasting insecticide treatment kit (ICON^®^ Maxx) against mosquitoes on various polymer materials

**DOI:** 10.1186/s12936-021-03909-6

**Published:** 2021-09-28

**Authors:** Patrick K. Tungu, Wema S. Sudi, Harparkash Kaur, Stephen M. Magesa, Mark Rowland

**Affiliations:** 1 Amani Medical Research Centre, National Institute for Medical Research, P.O. Box 81, Muheza, Tanzania; 2 London School of Hygiene and Tropical Medicine, London, WC1E 7HT UK; 3 Pan-African Malaria Vector Research Consortium, Muheza, P.O.Box 81, Tanga, Tanzania

**Keywords:** Insecticide-treatment kit, *Anopheles gambiae*, Malaria, Lambda-cyhalothrin, Polyester, Polyethylene, Cotton, Nylon, LLIN

## Abstract

**Background:**

Long-lasting efficacy of insecticide-treated nets is a balance between adhesion, retention and migration of insecticide to the surface of netting fibres. ICON^®^ Maxx is a twin-sachet ‘home-treatment kit’ of pyrethroid plus binding agent, recommended by the World Health Organization (WHO) for long-lasting, wash-fast treatment of polyester nets. While knitted polyester netting is widely used, fine woven polyethylene netting is increasingly available and nets made of cotton and nylon are common in Africa and Asia. It is important to investigate whether ICON Maxx is able to fulfill the WHO criteria of long-lasting treatment on a range of domestic fabrics to widen the scope for malaria protection.

**Methods:**

This study was a controlled comparison of the bio-efficacy and wash-fastness of lambda-cyhalothrin CS, with or without binder, on nets made of cotton, polyethylene, nylon, dyed and undyed polyester. Evaluation compared an array of bioassays: WHO cone and cylinder, median time to knockdown and WHO tunnel tests using *Anopheles* mosquitoes. Chemical assay revealed further insight.

**Results:**

ICON Maxx treated polyethylene and polyester netting met the WHO cone and tunnel test bio-efficacy criteria for LLIN after 20 standardized washes. Although nylon and cotton netting failed to meet the WHO cone and cylinder criteria, both materials passed the WHO tunnel test criterion of 80% mortality after 20 washes. All materials treated with standard lambda-cyhalothrin CS without binder failed to meet any of the WHO bio-efficacy criteria within 5 washes.

**Conclusion:**

The bio-efficacy of ICON Maxx against mosquitoes on netting washed up to 20 times demonstrated wash durability on a range of synthetic polymer and natural fibres: polyester, polyethylene, nylon and cotton. This raises the prospect of making insecticide-binder kits into an effective approach for turning untreated nets, curtains, military clothing, blankets—and tents and tarpaulins as used in disasters and humanitarian emergencies—into effective malaria prevention products. It may provide a solution to the problem of reduced LLIN coverage between campaigns by converting commercially sourced untreated nets into LLINs through community or home treatment. It may also open the door to binding of non-pyrethroid insecticides to nets and textiles for control of pyrethroid resistant vectors.

## Background

Insecticide-treated mosquito nets (ITNs), developed during the 1980s proved highly effective in reducing malaria-related morbidity and mortality [1]. Operationally, however, ITNs suffered several challenges in the field; these included the logistical problem of having to retreat nets every 12 months, the recurrent cost of annual retreatment and the unavailability of insecticides in remote places [2].

The advent of long-lasting insecticidal nets (LLIN) that do not require insecticide retreatment over a 3-years’ lifespan provided a technical solution to the logistical challenge of low retreatment coverage [3–6]. LLIN have since become the essential tool for vector control and malaria prevention in sub-Saharan Africa. The World Health Organization (WHO) recommends and promotes universal coverage of 1.0 LLIN for every 1.8 persons in populations at risk in malaria endemic countries [7]. The push towards this target has led to increased demand for LLIN by national malaria control programmes, international malaria control agencies and institutional buyers who have increasingly opted for LLIN as their preferred choice of malaria prevention [2, 8].

Thus far, international malaria control agencies have spent over two billion dollars on the provision of LLINs, leading to scale-up of access, which currently exceeds 50% of the population of sub-Saharan Africa [7]. The target of universal coverage is critical to success and while 50% is an impressive achievement, malaria elimination remains a distant prospect, and millions of African households remain unprotected particularly in the later stages, between universal coverage campaigns [9].

LLINs are treated with insecticide during net manufacture. However, the majority of ITN that are available through the commercial retail sector are not LLIN [9] and those nets in use, sourced from retail outlets, have either never been treated or were treated only once at the time of purchase [9, 10]. Locally sourced nets, which are not LLIN, may lose efficacy prematurely, long before the nets physically perish from wear and tear [9, 10]. This raises a need for treatment kits that can convert these nets post-manufacture into long-lasting insecticidal nets through simple household or community dipping.

Progress has been made with long-lasting treatment kits that can transform untreated nets into long-lasting treated nets by combining a conventional insecticide with a binding agent and the simple act of immersion into aqueous solution of the mixture. With this technology the untreated or conventionally treated nets already in use may be transformed into LLINs by the community post-manufacture under field conditions.

ICON Maxx is a long-lasting insecticide formulation developed by Syngenta in kit form [11]. Thus far, ICON Maxx is the only long-lasting insecticide treatment kit that has full recommendation of the World Health Organization for use on polyester nets [12, 13]. The kit is based on a slow-release capsule suspension (CS) formulation of lambda-cyhalothrin previously evaluated by the WHO and recommended for treatment of mosquito nets. ICON Maxx is presented as a twin sachet pack, containing lambda-cyhalothrin 10CS and binding agent, sufficient for the treatment of an individual mosquito net. The target dose of ICON Maxx on a family-size polyester mosquito net is 62 mg AI/m^2^. The actual dose received depends on the net size and can range from 50 mg AI/m^2^ (for a large family-size net) to 83 mg AI/m^2^ (for a single-size net). Efficacy and wash fastness of ICON Maxx has been demonstrated in several laboratory, experimental hut and field trials [14–16]. In all these studies the demonstration was made on nets made of polyester netting [14–17]. Although polyester is currently more widely used [15] it is not the only polymer used. Use of polyethylene nets is increasing, and nets of fine polyethylene weave are now available. Mosquito nets made traditionally from cotton are also common in countries of West Africa and South Asia. The global local retail market for cotton nets remains high. It is estimated that over 50% of nets sold in Iran and Pakistan are made of cotton. Nylon nets are used in India and Africa. There is also great diversity in the fabrics, and synthetic polymers used in curtains, blankets and other barriers to mosquitoes that are potentially treatable in the home.

The question is whether binder formulations can make these other types of polymer, aside from polyester, long-lasting. The efficacy and wash resistance of ICON Maxx needs to be confirmed on nets made of cotton, nylon, polyethylene and other synthetic materials before this product can have the widest possible application or impact on malaria.

Polyester and other netting materials come in a range of colours. There is some evidence that dye may affect the uptake and retention of conventional insecticide formulations during immersion [15]. It is important to confirm that uptake and retention of insecticide-plus-binder is not adversely affected by textile finishing.

The present study reports on the laboratory evaluation of bio-efficacy and wash-fastness of ICON Maxx on netting made of cotton, polyethylene, nylon, white and dyed polyester nets. This was done in controlled comparison with the same netting materials conventionally treated with lambda-cyhalothrin CS, a microencapsulated formulation (‘Iconet’, Syngenta UK) that does not include the long-lasting binder component.

## Methods

### Netting and treatment

Polyester white, polyester blue, polyethylene, cotton and nylon netting materials were used as substrates. Cotton nets were sourced from a manufacturer in Pakistan that supplies the national army, the polyethylene and nylon nets were sourced from manufacturers in India and the polyester nets were supplied by Vestergaard Frandsen. The absorbency of each material was determined using a test solution of ICON Maxx in de-ionized water. Solutions of ICON Maxx were specially prepared to match each material’s absorbency to achieve a similar target loading dose per unit surface area of 62 mg/m^2^ for ICON Maxx and 15 mg/m^2^ for Iconet. The nets were considered treated when all solution had been absorbed and all areas of the net were visibly wet without any dripping. The nets were dried horizontally in a darkened room at 30 °C on polythene sheeting and turned over every 10 min until completely dry. Each material was then cut into five 60 cm × 40 cm samples. Various positive and negative controls were introduced. Untreated samples of each material were retained as negative controls. Netting of each material treated with lambda-cyhalothrin 2.5% CS (Iconet), without binder formulation, served as positive controls for the ICON Maxx treated materials. ICON Maxx treated polyester white was used as the reference arm as it had already received recommendation by the WHO [12, 13].

### Washing procedure

Samples of each material were washed 0, 5, 10, 15 or 20 times. All samples were washed as 60 cm × 40 cm pieces except the polyethylene which was stiffer and harder to immerse and had to be cut into two to ensure thorough washing. The standard WHO Phase I laboratory washing procedure was adopted [14]. A soap solution of 2 g/l was produced using the soap Savon de Marseille and de-ionized water. Each net was placed in a 1 l bottle and immersed in 500 ml of soap solution before placement in a water bath. All samples were shaken at a rate of 155 movements per minute and remained immersed at 30 °C for 10 min. Each swatch was rinsed twice in de-ionized water under the same water bath conditions. A piece of each treated material was kept unwashed to serve as the zero-washed sample. Washing started on 20-wash pieces 20 working days (4 working weeks) before testing was due to start, on 15 wash pieces 5 days later, down to 5-wash pieces 5 days before pieces were due to be tested in rotation.

### Mosquitoes

All mosquitoes used were insectary reared non-blood fed female pyrethroid susceptible *Anopheles gambiae *sensu stricto (*s.s*.) (Diptera: Culicidae) mosquitoes (Kisumu strain), susceptible to all pyrethroids, reared in the National Institute for Medical Research, Ubware Centre. Pyrethroid susceptible mosquitoes were used as these were most sensitive for showing changes in binding affinity of the formulations on polymers over multiple washes. Pyrethroid resistant mosquitoes are less sensitive/suitable for demonstrating pyrethroid-binder retention/loss when using mortality/or knockdown is the outcome measures.

### Cone bioassays

To evaluate the efficacy of ICON Maxx and Iconet treated netting materials, standard WHO cone bioassays were performed, based on the WHO Phase I protocol [14] against insectary-reared pyrethroid-susceptible *An. gambiae* Kisumu strain. Four WHO cones were fixed to each netting sample and 5 mosquitoes aged 2–5 days old were introduced into each cone. After 3 min exposure the mosquitoes were transferred to holding cups. Control mosquitoes were exposed to untreated netting. The 20 mosquitoes tested per replicate were provided with a pad of glucose solution for nourishment. Tests were done at 25 °C and 70% RH. Knock-down (KD) was recorded 1-h post-exposure and mortality 24 h later. Five replicates were carried out per sample, 100 mosquitoes per treatment. If control mortality exceeded 10% on any day the results were discounted and the test repeated; this procedure was followed in all bioassay tests described. All replicates of the various textile-wash treatments were carried out in strict rotation using Latin squares to adjust for any variation in insect batch or test conditions.

### Cylinder bioassays

In preparation for this assay, treated and washed samples of each material were cut and stapled to pieces of plain paper measuring 12 cm × 15 cm before insertion inside WHO susceptibility test cylinders and securing with metal rings. Ten 2–3-days old female mosquitoes were introduced to each holding chamber and transferred into the test chamber where they were exposed for 3 min. After exposure, the mosquitoes were returned to the holding chambers and given access to sugar solution. The number knocked down was recorded after 60 min and the number dead was recorded 24 h later. A negative control of mosquitoes exposed to untreated netting material was carried out in parallel each test.

### Median time to knock down (MTKD)

In the MTKD bioassay eleven mosquitoes 2–3 days old were introduced into a WHO wire ball frame covered with the treated material [14]. Knock down was defined as a mosquito lying either on its back, side or no longer able to support itself. The time taken for each mosquito to knockdown was recorded up to the median (6th) mosquito. Nine replicates were carried out for each treatment, material and wash number, using Latin squares. Untreated net of each material was used as a negative control.

### Tunnel tests

Tunnel tests were used to assess unwashed treated netting and netting washed 20 times as a proxy for 3 years of field use, as per WHO guidelines [14]. Samples were selected at random and not according to whether they had passed or failed the cone test. The tunnels consisted of three chambers, the mosquito release chamber C1, a middle chamber C2, and the baited chamber C3 containing a caged guinea pig separated from chamber C2 by the test netting. Test netting was fitted to a rectangular frame measuring 25 cm × 25 cm, which slotted across the tunnel between chambers C2 and C3. Nine holes 1 cm in diameter were cut in three rows of three through which host-seeking mosquitoes could penetrate. C1 and C2 were separated by a paper screen with a single 9 cm diameter hole through which host-seeking mosquitoes attracted by the bait odour from C3 could fly from C1 to C2 and from thence to C3 via the 9 holes. This arrangement encouraged exposure through the net while actively bait seeking rather than passively resting on the treated net during the dusk period. A total of 100 susceptible 5–8-day old mosquitoes were tested in two replicates of 50 mosquitoes. Mosquitoes were introduced to C1 in late afternoon and left until the following morning, 15 h later, when the mosquitoes in each chamber were counted and their status, alive or dead and fed or unfed, were recorded. The live mosquitoes were kept in a paper cup with access to a sugar solution for a further 24 h after which delayed mortality was scored. Two replicates of each material were carried out. A negative control test of untreated material was tested on each day; a replicate was only considered eligible if in the negative control a minimum of 50% blood-fed. Nettings were tested at random within latin squares until all replicates were completed; a negative control was included on each test day. Mortality was control-corrected.

### Chemical analysis

High pressure liquid chromatography HPLC was used to determine the concentration of insecticide on each treated piece of net after washing the requisite number of times. Four pieces measuring 5 × 5 cm of each net treatment were cut and placed in a borosilicate glass vial with 1 ml of acetronitrile. The vials were sonicated for 10 min; the solution was removed and placed in HPLC vials. The HPLC analysis was carried out at the London School of Hygiene and Tropical Medicine using a Dionex Summit range of equipment and software (Camberley, Surrey, UK). The samples were separated using an AcclaimR C18 120 (250 × 4.6 mm, Dionex, UK) column eluted with water/acetonitrile (90:10%; v/v) at a flow rate of 2 ml/min and passed through the photodiode array detector (PDA-100, Dionex) set at 27 nm. The authenticity of the detected peaks was determined by comparison of retention time, spectral extraction at 275 nm and spiking the sample with commercially available standards.

### Statistical analysis

Mixed effects generalized linear models using STATA^®^ 15 (Stata Corporation, Collage Station, TX, USA 2005) were used for analysis. The independent variables included treatment (ICON Maxx, Iconet), net material (the 5 types of polymer), number of washes (0, 5, 10, 15, 20 for Icon Maxx or 0, 5, 10 for Iconet), number of replicates adjusting for group size, and the interactions between net type and number of washes. Mixed effects linear regression was used to analyse the content of lambda-cyhalothrin in netting samples (AI retention index) extracted by HPLC, adjusting between netting materials, treatments and number of washes. Mixed effects logistic regression models were used to analyse the change in biological responses (proportions killed, knocked down) after washing of materials, and testing with cone, cylinder or tunnel tests.

### Ethical clearance

Approval was obtained from the ethics committees of the London School of Hygiene and Tropical Medicine and the Tanzanian National Institute of Medical Research (Ref: NIMR/HQ/R.8a/Vol. X/86). The procedure for use of guinea pigs in tunnel tests conformed to criteria established in EC Directive 86/609/ECC regarding protection of animals used for experimental purposes.

## Results

### Chemical analysis

**Fig. 1:**
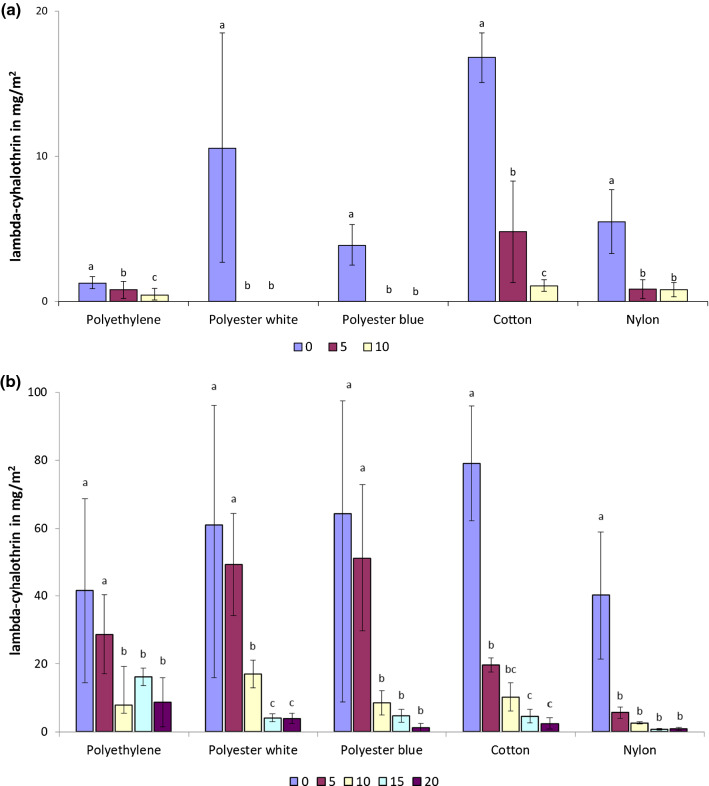
**a** Mean lambda-cyhalothrin content (± 95% CI) for netting materials treated with Iconet and washed up to 10 times. **b** Mean lambda-cyhalothrin content (± 95% CI) for netting materials treated with ICON Maxx and washed up to 20 times

Despite applying the target dose of 15 mg/m^2^ lambda-cyhalothrin, chemical analysis by HPLC of the Iconet CS treated materials (without binder) showed that cotton and undyed polyester (white) had higher affinity for lambda-cyhalothrin CS as compared to dyed polyester (blue), nylon and polyethylene (mixed effects linear regression F_(4, 15)_ = 26.5, p = 0.005). Within 5 washes almost all detectable lambda-cyhalothrin was removed from the two polyester nettings and less than 1 mg/m^2^ was detectable on polyethylene and nylon. More insecticide (4.8 mg/m^2^) was retained in cotton fibres than in other nettings at 5 washings (Mixed effects linear regression F_(4, 15)_ = 207.9, p = 0.0001) (Fig. 1a).

**Fig. 2:**
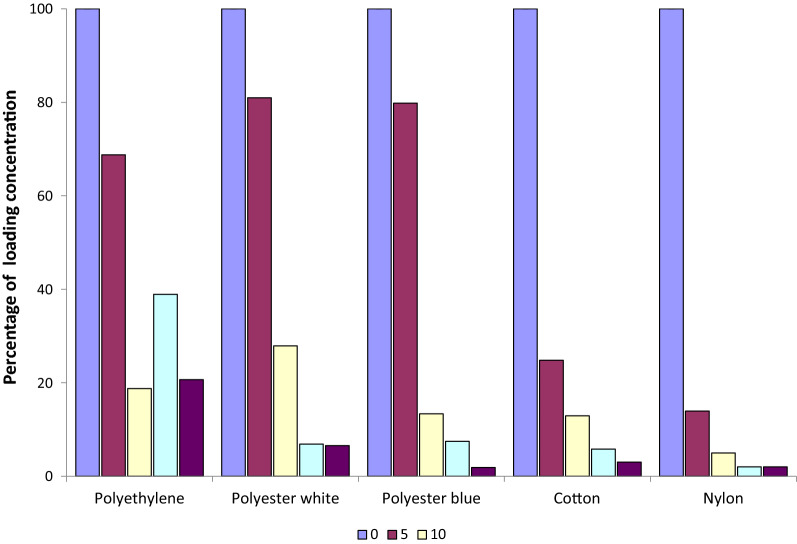
Content of lambda-cyhalothrin as a percentage of the ICON Maxx loading dose over 20 washes

Chemical analysis of the ICON Maxx treated materials (with binder) showed that all materials had more higher affinity for the pyrethroid with binder than for pyrethroid without binder as compared with the Iconet formulation (Mixed effects linear regression, F_(1, 38)_ = 60,8, p = 0.0001). Within the ICON Maxx treatments, cotton and polyester undyed and dyed (white and blue) showed higher affinity or absorption (79, 61, 64 mg/m^2^, respectively), with loading dosages of lambda-cyhalothrin similar to the target dose of 62 mg/m^2^, whilst polyethylene and nylon showed lower loading dosages of only 42 and 40 mg/m^2^, well below the intended target (Fig. 1b). After washing 0–5 times, the two polyesters and the polyethylene showed particularly high retention of insecticide, with over 70% of the initial lambda-cyhalothrin content remaining (Fig. 2): none of these 3 materials showing a significant decline in content after 5 washes (Fig. 1b). The mixed effects linear regression showed that the loss of insecticide was significantly greater in cotton (t = − 17.0, p = 0.001) and nylon (t =  − 9.2, p = 0.001) at 5 washes (Fig. 1b) with neither material retaining more than 25% of loading dose (Fig. 2). Comparing polyester blue and polyester white over all 0–20 washes there was no evidence that polyester blue showed less affinity for ICON Maxx on impregnation or retained less AI than polyester white over 0–20 washes (t = 0.20, p = 0.845) (Figs. 1b, 2) in the mixed effects linear regression. The only material of the 5 tested in which binding and retention of AI over 20 washes was significantly less than the other materials in the analysis was nylon (t =  − 3.69, p = 0.001).

### Cone bioassays

#### Iconet treated materials

**Fig. 3:**
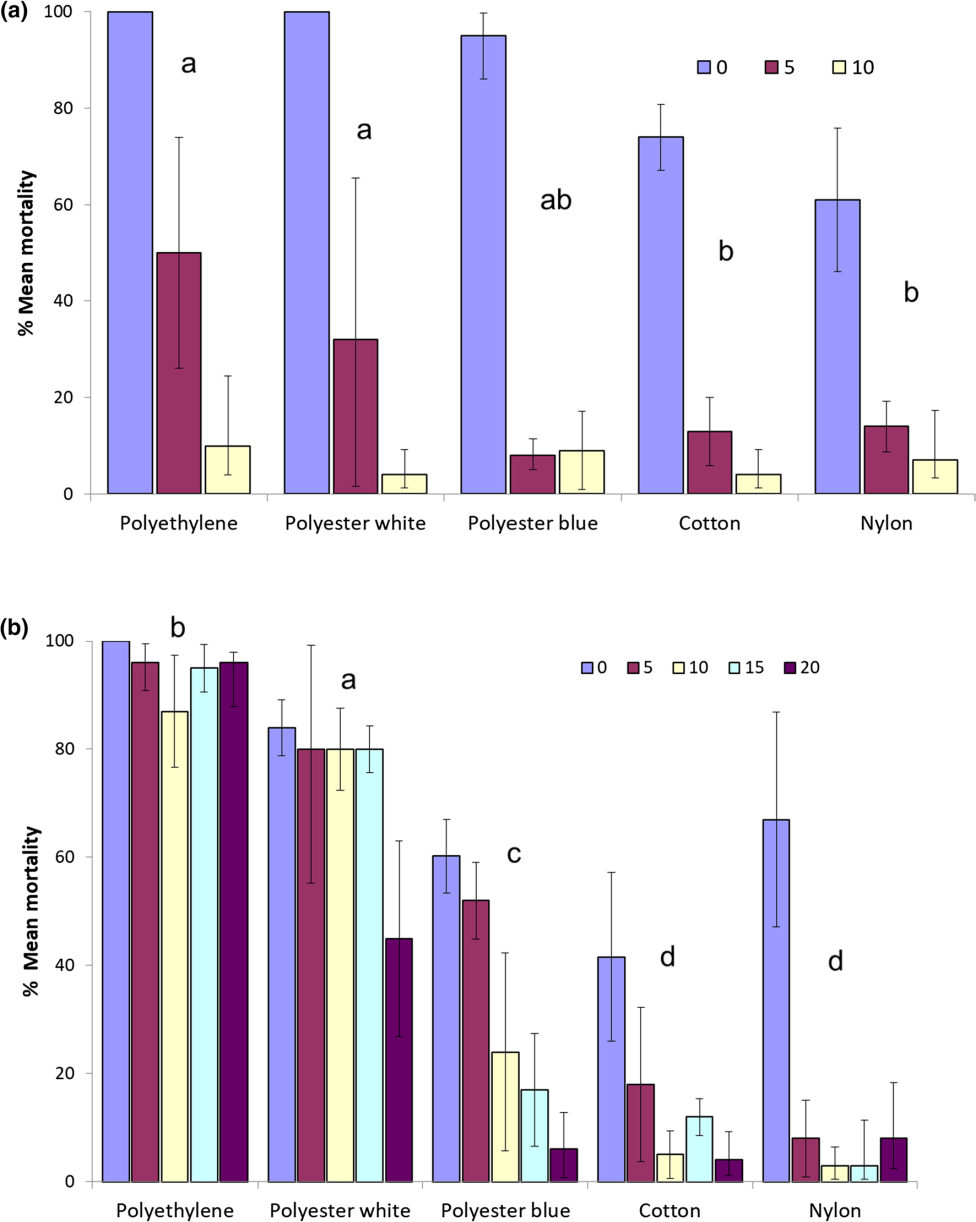
**a** Cone bioassay: Percentage mortality (± 95% CI) at 24 h after exposure for netting materials treated with Iconet and washed up to 10 times. **b** Cone bioassay: Percentage mortality (± 95% CI) at 24 h after exposure for netting materials treated with ICON Maxx and washed up to 20 times

The mosquito mortalities induced by Iconet treated polyester white, polyester blue and polyethylene in cone tests all exceeded 90% after loading, whilst cotton and nylon only induced between 60 and 80% mortality after treatment (Fig. 3a). Comparing all materials (using mixed effects logistic regression, adjusting for sample size in replicate tests), polyethylene and polyester white recorded higher mortality than other materials across the first 5 washes (z = − 1.75, p = 0.001). Mortality decreased to less than 10% after 10 washes across all materials. The knockdown trend was consistent with the mortality trend (Table 1).

**Table 1:** Cone bioassays: percentage knockdown at 60 min after exposure to netting materials treated with Iconet or ICON Maxx and washed up to 10 times or 20 times, respectively

Washes	Polyethylene	Polyester white	Polyester blue	Cotton	Nylon
Iconet					
0	100	100	90	100	96
5	70	56	14	13	9
10	40	15	5	4	0
IconMaxx					
0	98	72	90	65	100
5	99	70	81	39	76
10	64	72	36	6	22
15	99	66	24	15	0
20	99	39	1	2	3

#### ICON Maxx treated materials

The mortality induced by ICON Maxx treated polyethylene netting in cone bioassays was 100% after loading and exceeded 90% mortality after 20 washes. Mortality on polyethelene was significantly higher than on all other materials across each wash point including polyester white (z = 5.8, p = 0.001). Mortality induced by polyester white exceeded 80% over 0–15 washes decreasing to 45% only after 20 washes. Mortality induced by polyester blue was only 60% at loading decreasing to 24% at 10 and 6% at 20 washes, thus confirming the poorer adhesion and retention of the binder formulation on dyed compared to undyed polyester (z = − 10.6, p = 0.001). Mortality of ICON Maxx on cotton and nylon while initially efficacious was not sustained after 5 washes, and retention on these materials was lowest of all (z = − 14.3, p = 0.0001).

The knockdown trend was consistent with mortality; polyethylene recorded higher knockdown than any other material, followed by polyester white and then polyester blue. Percentage knockdown on treated cotton and nylon decreased after 10–15 washes (Fig. 3b; Table 1).

### Cylinder bioassays

#### Iconet treated materials

As in cone tests, the highest mortality recorded in cylinder tests with Iconet treated netting was with polyethylene, polyester white, polyester blue and cotton, followed by nylon (71%). Within 5 washes efficacy was inadequate.

**Fig. 4:**
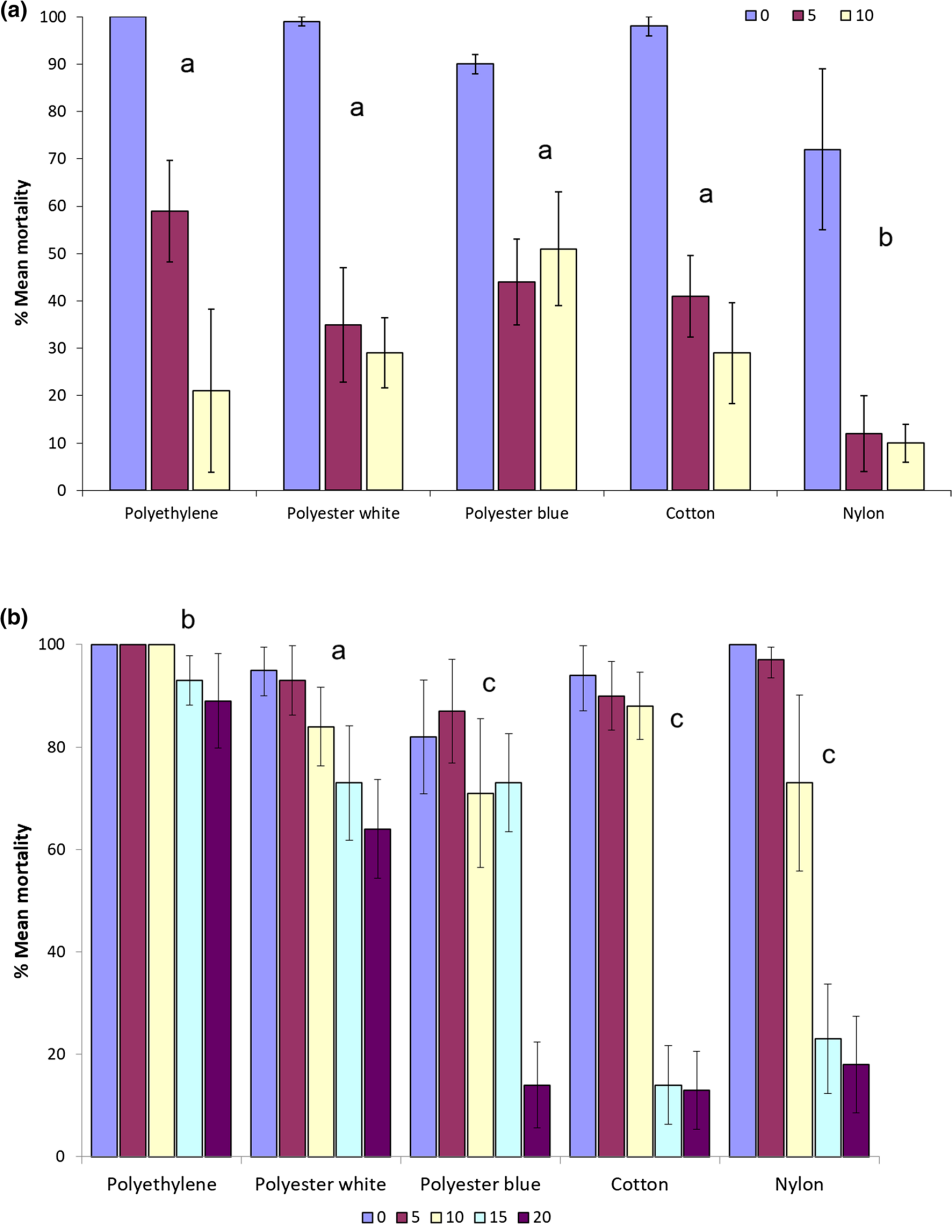
**a** Cylinder bioassay: Percentage mortality (± 95% CI) at 24 h after exposure for netting materials treated with Iconet and washed up to 10 times. **b** Cylinder bioassay: Percentage mortality (± 95% CI) at 24 h after exposure for netting materials treated with ICON Maxx and washed up to 20 times

With respect to knockdown, all unwashed materials recorded 100% knockdown at 60 min except nylon at 85%. However, at 5 washes, percentage knockdown on all materials except polyethylene had decreased to 50% or less (Fig. 4a; Table 2).

**Table 2:** Cylinder bioassays: percentage knockdown 60 min after exposure to netting materials treated with Iconet or ICON Maxx and washed up to times or 20 times respectively

Washes	Polyethylene	Polyester white	Polyester blue	Cotton	Nylon
Iconet					
0	100	100	100	100	85
5	70	15	29	50	6
10	38	1	25	35	1
IconMaxx					
0	100	100	97	99	100
5	100	100	97	99	100
10	100	93	94	97	75
15	100	94	85	49	20
20	100	88	10	35	21

### ICON Maxx treated materials

With all netting materials, cylinder mortality was exceptionally high (> 95%) after treatment (0 washes) and also at 5 washes. Polyethylene recorded significantly higher performance than all other materials with 100% mortality at 0, 5 and 10 washes and was the only material to exceed 80% mortality at 20 washes (z = 8.18, p = 0.0001). Polyester white and cotton exceeded 80% mortality at 10 washes. Polyester blue exceeded 70% at 15 washes. Polyester white recorded significantly higher mortality than polyester blue from 0 to 20 washes (z = 4.34, p = 0.001). Polyester white decreased below 80% after 15 washes and blue after 10 washes. Mortalities recorded with nylon and cotton decreased below 20% at 15 washes (Fig. 4b). The only materials that incurred loss of activity at 15 washes were cotton and nylon.

With respect to 60-min knockdown, every material recorded between 100 and 97% knockdown after 0 and 5 washes. After 20 washes, only polyethylene showed 100% knockdown and only polyester white recorded 80% knockdown. Knockdown on cotton and nylon showed decrease between 10 and 15 washes (Table 2).

### ICON Maxx comparative efficacy in cone and cylinder bioassays

**Fig. 5:**
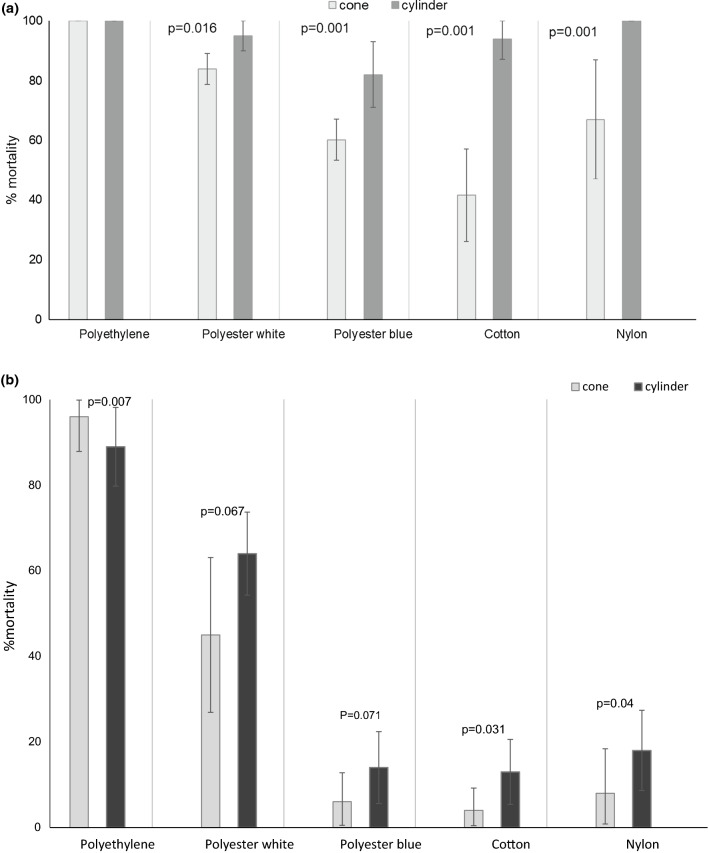
**a** Cone and cylinder bioassay comparison: percentage mortality (± 95% CI) at 24 h after exposure for polyester white nets treated with ICON Maxx at 0 wash point. **b** Cone and cylinder bioassays comparison: % mean mortality at 24 h post exposure (± 95% confidence intervals) for polyester white nets treated with ICON Maxx at 20 wash point

Comparing all treated materials, higher mortality was recorded in the cylinder bioassay as compared to the cone bioassay at each wash point (mixed effects logistic regression, z = 10.6, p = 0.001) (Figs. 5a, b). As exposure time was the same in cone and cylinder, this higher mortality was probably due to a higher ratio of netting covered surface to uncovered plastic surface in the cylinder as compared to the cone. Mortality was consistently high in the cylinder at 0 washes (> 80% mortality) for each material tested compared to the cone (Fig. 5a). At 20 washes the difference in mortality between cylinder and cone was smaller and yet consistent for each material. The difference in mortality between cone and cylinder was due to variation in AI retention between the 5 materials (z = 4.8 to 9.0, p = 0.001) in addition to differences in efficacy between cylinder and cone (z = 10.6, p < 0.001) (Fig. 5b).

### Tunnel tests

With the unwashed ICON Maxx treated materials, blood-feeding inhibition was higher than the WHO 90% threshold with polyethylene, polyester white and polyester blue. Percentage mortality with all 5 materials ranged from 91 to 96%, well above the 80% threshold. After 20 washes, polyethylene, nylon and polyester white all passed the WHO criterion of 90% blood-feeding inhibition (Table 3). Percentage mortality with polyethylene, cotton and nylon ranged ranged from 83 to 88%; hence all these polymers passed the WHO mortality criterion. Mortality was less than the 80% threshold with only polyester white (the reference net) and polyester blue.

**Table 3:** Tunnel tests: percentage passage, blood-feeding inhibition and mortality after exposure to ICON Maxx treated netting at 0 and 20 washes

Number of washes	Material	Passage inhibition %	Blood-feeding inhibition %	Mortality %
0	Untreated net	56	–	0
	Polyethylene	57	90	95
	Polyester white	82	100	96
	Polyester blue	64	93	91
	Cotton	37	82	94
	Nylon	16	100	94
20	Polyethylene	29	90	86
	Polyester white	22	100	54
	Polyester blue	25	86	65
	Cotton	24	75	83
	Nylon	14	100	88

Comparing cylinder and tunnel at 0 washes, all materials recorded well over 90% mortality in both assays. At 20 washes (the critical threshold) all materials (except polyethylene) recorded below 20% mortality in cone and cylinder and yet all materials passed the tunnel test with the exception of polyester white and blue. Not surprisingly perhaps, there was no association between the results of tunnel tests and the results of cone and cylinder tests; the only exception was polyethylene which passed the thresholds of all the bioassays.

### Median time to knock down bioassays

Median time to knock down (MTKD) is considered a good indicator of surface AI. By noting the median responder in an MTKD assay and running several replications, it is possible to generate the confidence interval around the mean of the medians. MTKD with all unwashed Iconet treated materials were statistically similar with the exception of nylon, which took 1.5–2 times longer to reach than other materials. After 5 washes and 30 min testing, no Iconet treated materials reach median knockdown.

With all ICON Maxx treated materials, the difference in MTKD between 0 and 5 times washed materials did not differ significantly; thus the binder was retaining the lambda-cyhalothrin on the netting surfaces (Table 4). With polyethylene white and polyester blue, MTKD was not showing significant differences between 0 and 10 washes, although there was some indication of MTKD taking longer to reach 10 washes. With cotton and nylon, MTKD was not reached within 30 min exposure on 10-times washed netting indicating loss of bioavailability of surface AI on these samples (Table 4).

**Table 4:** Median time to knock down in minutes (95% CI) on ICON Maxx treated materials after 0, 5 and 10 washes

Material	0 washes	5 washes	10 washes
Polyethylene	10^a,1^ (0.9)	11.8^a,1^ (0.9)	14.2^a,2^ (0.6)
Polyester white	14.6^b,1^ (0.6)	13.7^b,1^ (0.9)	14.7^a,1^ (0.6)
Polyester blue	9^a,1^ (1.3)	10.3^a,1^ (1.6)	14.1^a,2^ (0.8)
Cotton	14.5^b,1^ (0.8)	14.1^b,1^ (0.8)	> 30^c,2^ (0)
Nylon	10.3^a,1^ (1.1)	14.2^b,2^ (0.7)	> 30^c,3^ (0)

Within each wash-point column, materials sharing same letter superscripts do not differ statistically (p ≥ 0.05). Within each wash-point row, materials sharing same numeric superscripts do not differ statistically (p ≥ 0.05)

Regression analysis showed surface content of insecticide had significant effect on MTKD; for every 1 mg decrease in insecticide content there was 3 s increase in MTKD (F_1,50_ = 6.27, p = 0.0156).

## Discussion

There were four objectives. The primary objective was to determine whether the pyrethroid lambda-cyhalothrin bound within a polymer resin could improve the wash fastness of the insecticide on nets made from a range of synthetic polymers, natural fibres and dye finishes. To complement the study, controlled comparison was made with a standard pyrethroid CS treatment which lacked the binder. The second objective was to take the treatments through the WHO LLIN evaluation process to determine which insecticide-treated substrates would withstand 15–20 washes and potentially achieve WHO recommendation. The third objective was to introduce new types of bioassays and compare against the cone test to test their potential utility. The fourth, but not least, was to consider some currently neglected humanitarian contexts badly in need of vector borne disease control and consider whether insecticide-binder treated substrates could provide a solution.

According to WHO, LLIN evaluation guides, polyethylene, cotton and nylon treated with ICON Maxx met the tunnel test criteria of > 80% mortality after 20 washes in Phase I, which is a recognized surrogate for 3 years of pyrethroid durability on household LLIN [18]. Polyester white (undyed), the positive control, and polyester blue fell short of the WHO tunnel criteria in Phase I tests but elsewhere in other studies they did achieve the WHO threshold [16, 17, 19], emphasizing the importance of multiple trials before coming to a consensus conclusion. Polyethylene also met the required criterion of > 80% mortality in cone tests and was the best performing polymer of the four tested. In all bioassays except the tunnel test, cotton and nylon netting did not reach the WHO threshold. Most of the textiles performed well in one or more types of bioassays and none can be ruled out as a suitable substrate for vector control treatment. Even polyester white, the positive control, which failed to meet the required threshold in the Phase I, fared well in Phase II (experimental hut) trials and Phase III (field trials of insecticide durability) in the same locality [12, 13, 16]. Therefore, the Phase I tests reported here are best viewed comparatively, one textile versus another, rather than as pass or fail.

By contrast, none of the nettings—polymer or natural fibre—withstood more than a few washes, when treated with a standard lambda-cyhalothrin CS formulation. There is no question of the superiority of the binder formulation, which is a genuine technical advance for a variety of potential malaria control substrates or contexts [12].

### Nylon

Nylon showed poorer adhesion of ICON Maxx on loading and poorest wash retention of all materials, losing 84% of insecticide content within 5 washes and 98% within 20 washes. In cone bioassay, mortality decreased by 92% within 5 washes and yet in cylinder bioassay where most of the interior was netting-covered, mortality stood at 97% after 5 washes and only decreased to low level after 10–15 washes. In tunnel test, ICON Maxx treated nylon passed the WHO criterion of > 80% mortality at 20 washes. Of all the materials tested, nylon was the most unpredictable. While its efficacy in tunnel was encouraging, nylon failed as a substrate of preferred choice for ICON Maxx treatment due to the poorer absorption, adhesion and wash-resilience. If there is a choice of material, the better option would be substitution of nylon with a better adhering or wash-tolerant polymer. On ICON Maxx treated nylon net curtains, as a barrier against Aedes and for prevention of Aedes borne arboviruses, it may have potential, warranting further studies in household conditions.

### Cotton

Owing to the high absorptive property of cotton, the cotton samples contained the highest loading dose of ICON Maxx initially. However, as on nylon, adhesion and retention of the insecticide was poor, content decreasing by 75% after 5 washes and by 96% after 15 washes. Cone bioassay recorded only 40% mortality at 0 washes and 4% at 20 washes but, as was the case with nylon, mortality in cylinders was high between zero and 10 washes and only decreased sharply at 15 washes. As with nylon, cotton passed the tunnel test criterion for LLIN at 20 washes. The presence of a high dose of lambda-cyhalothrin together with a low insecticidal activity suggests that bioavailability on the surface of cotton netting fibres is low, that is, most of the insecticide remained locked within the cotton fibres and failed to make contact with mosquito tarsi. This was not the case with synthetic fabrics such as polyester and polyethylene on which the insecticide is readily bio-available on the surface of fibres. Other studies have also reported the low insecticidal property of pyrethroids on cotton as compared to other fabrics [20, 21]. However, with the tunnel test, results with cotton netting exceeded 80% mortality after twenty washes, so bringing cotton into line with WHO criteria for recommendation [14].

### Polyethylene

As with nylon, polyethylene treatment demonstrated a relatively low loading dosage (40 mg/m^2^) but in contrast to nylon and cotton, polyethylene showed better retention at 5 washes and a more regular loss rate over the course of 0–20 washes. Mortality in cone and cylinder bioassay was consistently high (~ 95%) over the course of 20 washes, and thus a completely different trajectory compared with nylon and cotton. ICON Maxx seemed to stay bound to the polyethylene, which remained fully toxic whereas the binder seemed lost from nylon and cotton during washing. As with nylon and cotton, polyethylene exceeded the tunnel test criteria at 20 washes.

Polyethylene seems an ideal substrate for ICON Maxx. In some studies, polyethylene netting materials were shown to be strong and able to tolerate five years of field use [3, 22]. More recently in larger scale surveys polyethylene has shown poor durability [23], somewhat improved by changing the knitting weave [23].

### Polyester

The superiority of ICON Maxx on polyethylene compared to undyed polyester white was a surprise since the latter was the positive control and the polymer netting that ICON Maxx was designed for originally. While both polyester white and blue fell consistently short of polyethylene in an array of bioassay tests, ICON Maxx did attain WHO recommendation for use on polyester over 15–20 washes which is a significant increase in wash-tolerance compared to the standard CS formulation tested in this paper. After Phase II (experimental hut) trials and Phase III (three-year field trials of insecticide durability) ICON Maxx did attain WHO recommendation as a polyester long-lasting treatment [13]. Comparing undyed and dyed polyester netting, the chemical analysis indicated similar loading dosages, implying that the binder in ICON Maxx had largely overcome the poor adherence induced by the dye of earlier formulations on polyester [18]. At most wash points the rate of loss of insecticide was similar between polyester blue and polyester white treated with ICON Maxx. While polyester white tended to record greater mortality than polyester blue in some bioassays, the differences were marginal and not consistent between all types of bioassays.

### Comparison of ICON Maxx with KO-Tab 123

ICON Maxx is not the first wash-resilient formulation to be developed [12]. KO-Tab 123 was a wash-resilient formulation of deltamethrin (25 mg/m^2^) and binder rather than lamba-cyhalothrin (55 mg/m^2^) and binder in ICON Maxx [24]. Its development coincided with the development of factory produced LLIN and it was not taken forward to Phase III field trials. Had it done so, it might have proven as effective as ICON Maxx, which did go on to Phase III evaluation and obtained WHO full recommendation for 2.5–3 years of effective field use [12]. When compared with ICON Maxx on the same materials as tested in the present paper, it showed similarity in characteristics over 20 washes: high insecticide retention and bio-efficacy on undyed polyester and polyethylene and poorer retention and bio-efficacy on cotton and nylon [25].

### Choice of testing methodology: cone, cylinder or tunnel

Despite having the same 3-min exposure, the mortality/knockdown responses differed considerably between cone and cylinder tests. The purpose of the comparison was to identify whether the cylinder should supplant the cone as the primary WHO insecticide bioassay. Both are WHO bioassays. The cone bioassay was initially designed for assessment of IRS bio-efficacy and residual activity on hard flat wall and ceiling surfaces of sprayed houses. Only later was it re-purposed for use as an ITN/LLIN bioassay. The IRS bioassay exposes mosquitoes for 30 min; this gives a mortality similar to that of free-flying mosquitoes entering and exiting IRS sprayed experimental huts [26] and is, therefore, appropriate as an exposure time. For ITN testing the cone has limitations: contact time is shorter and it is difficult to ‘settle’ the mosquitoes on cone netting for the prescribed 3 min. If the purpose of a residual bioassay is to manage undesirable variables, then control of exposure time is essential in a short exposure assay. In this respect, the cylinder is an improvement over the cone; when cylinder and cone mortality are compared, mortality is higher in the cylinder than in the cone due to higher ratio of netting to plastic. This was particularly evident at zero washes. But is the cylinder any less variable than the cone? The mortality at 20 washes for the different polymers tested would suggest not. Mortality rose and fell between the cylinder and cone in synchrony depending to the attributes of the netting surface and AI concentration retention rather than with other attributes of the test method. If exposures longer than 3 min are required, for example when testing resistant strains, the cylinder would be the better, more precise tool to use than the cone.

On the other hand, if the aim is to simulate natural host-seeking behaviour on and around the net then the overnight tunnel test is the more realistic bioassay than either the cone or cylinder. In the 3 min cone or cylinder bioassay the mosquito is standing on the netting or flitting around it, whereas in the night-time tunnel test the host-seeking mosquito is trying the penetrate through the net. Different anatomical parts may be in contract with the net and for different lengths of time in the tunnel. The correlations between cone and tunnel and between cylinder and tunnel remain weak.

In these tests, susceptible mosquito strain was deliberately used as they are the most sensitive tool to investigate the properties of surface binder and pyrethroid over changing concentration before and after washing. In the wild of course, many mosquito populations will also contain insecticide resistant mosquitoes, and these may, or may not, be killed by the surface pyrethroid. If not killed, they may still be repelled or inhibited from blood-feeding. Eighty per cent of LLIN in use for malaria control are still standard pyrethroid-only LLIN and only a fraction will contain a second active ingredient. One of our aims was not just to facilitate treatment of untreated nets with a wash-tolerant pyrethroid but to encourage manufacturers to produce sachets of alternative active ingredients with which to make standard pyrethroid LLINs to become more effective ‘mixture nets’.

### Future uses

The obvious use for ICON Maxx and other treat-it-yourself long lasting pyrethroid kits is to bundle the sachets with the hundreds of thousands of untreated nets that continue to be sold in retail markets, rural and urban. Conical nets, at the ‘luxury’ end of the market are rarely bundled with kits, and the wholesalers and retailers of untreated conical and rectangular nets will need regular supplies of kits.

This is a timely reminder for beneficiaries of free distributions of LLIN, who may know little or nothing about LLIN production, that LLIN are special because of the insecticide they contain, and the nets need to be used with care and respect. Older nets can be made more protective with a top-up of insecticide, especially if the next universal coverage campaign is delayed, giving older nets a further 2–3 years of protective use. Universal campaigns are often supplemented with top-ups of new LLINs in the interval between campaigns, and if LLIN numbers are in short supply, untreated nets and older LLINs that are still serviceable would continue to provide benefit if re-treated.

Mosquito nets are not the only household product which might obtain benefit from long-lasting insecticide treatment. Curtains made of nylon, polyethylene or cotton could provide family protection from *Anopheles* vectors of malaria and *Aedes* vectors of dengue, chikungunya and yellow fever. These could be immersed in ICON Maxx solution like the netting described in this article, or sprayed with deltamethrin 62 SC-PE (polymer-enhanced suspension concentrate formulation), a product specially derived from KO-Tab-123 technology as an aqueous spray formulation (K-Othrine Polyzone, Bayer Crop Sciences, Germany) [28].

Armed services have favored the use of permethrin on combat clothing for personal protection because of its high repellence [29, 30]. Alphacyano-pyrethroids such as ICON Maxx might be preferred in certain locations due to its higher toxicity compared to permethrin. To prevent skin irritation the treated material might be separated from skin contact by a non-treated inner layer of material [31].

### Disasters and humanitarian emergencies

The same arguments apply to civilian bedding and to top-sheets and blankets treated and distributed in epidemics, disasters or emergencies [32, 33]. Standard issue in humanitarian emergencies are blankets, tents and polyethylene tarpaulins [31, 33, 34] particularly for refugee populations on the move, i.e. situations where nets are dysfunctional or where sprayable housing is absent. Acute phase emergencies are a niche, which has proven difficult to supply with adequate vector control protection. The problem is compounded by the sectorial nature of international aid. Blankets and tents in emergencies are administered by the shelter sector, vector control is administered by the health sector. Blankets, sheets and shelters are also location-specific, and utility and material will depend on climate and ambient temperature. The solution might be to coordinate the shelter and public health sectors to treat whatever shelter or material is provided on-site with a long-lasting insecticide or repellent formulation mixed with binder formulation and UV protectant, applied by immersion, spray pump, or treated at source during manufacture. Bespoke factory manufactured products may not justify the investment in stockpiling, bespoke long-lasting formulations that can used to treat a variety of products could, on the other hand, justify the investment and be shifted fast to where it is needed.

The treatment of polyethylene tarpaulins or shade cloth with pyrethroid plus binder as used in emergency shelter has formed the basis of the insecticide treated wall liner concept of protection in the home [35].

### Dual-AI LLIN and non-pyrethroid long-lasting treatment kits

The first Dual Active Ingredient LLINs were the PBO-synergist nets PermaNet 3.0 [36] and Olyset Plus [37]. Whilst the pyrethroid in all WHO recommended LLINs should remain effective for 3 years, WHO is now referring to Dual-AI nets as ITNs because it is not clear whether the PBO component will last a full 3 years of field use [38]. Whilst Olyset Plus, the first in class pyrethroid–PBO net, has demonstrated effectiveness for two years, it is not yet clear in the ongoing cluster randomized trial whether the PBO will remain effective for the full 3 years. If it falls short of 3 years, there is an opportunity here to apply PBO via a PBO-binder long-lasting kit after 2 years to take it through the third year. Similarly, there is an opportunity for a PBO-binder long-lasting kit to be applied to any pyrethroid LLIN to convert those to pyrethroid-PBO LLIN. This could apply equally to other partner AI, such as pyriproxifen or chlorfenapyr which are being used with pyrethroid in other types of Dual-AI LLIN should these fall short of 3 years’ effectiveness [39]. In environments with high pyrethroid resistance, it would be a mistake to allow Dual AI nets to revert to a pyrethroid-only LLIN in their third year as users would be only be part-protected.

## Conclusion

In all tests performed, ICON Maxx treated polyethylene recorded greater performance than the positive control (polyester white) and other netting materials tested. Although the efficacy of ICON Maxx on cotton and nylon netting were low compared to other materials, they still met WHO criteria for LLIN. All ICON Maxx treated materials demonstrated insecticidal efficacy after twenty washes and met WHO criteria for long-lasting insecticidal treatment in one or more bioassays described here. Chemical analysis confirmed that lambda-cyhalothrin was more strongly retained in the ICON Maxx-treated than in Iconet treated materials. The high efficacy, wash-fastness and versatility of ICON Maxx raises the prospect of it becoming an all-purpose formulation for such purposes as military clothing, civilian bed covers and curtains, or for blankets, tarpaulins and tents distributed in epidemics, disasters or humanitarian emergencies, rather than dream of bespoke long-lasting insecticidal products for niche markets that may not be viable investment for manufacturers. ICON Maxx or treatment kits like ICON Maxx may provide an answer to the problem of reduced LLIN coverage between distribution campaigns, by turning commercial retail-sourced untreated nets into LLINs through simple home or community treatment.

## Data Availability

The datasets used and/or analyzed during the current study are available from the corresponding author on reasonable request.
